# Enhanced Tolerability and Improved Outcomes in Acne Management: A Real‐World Study of Dermocosmetic Adjunctive Therapy

**DOI:** 10.1111/jocd.16772

**Published:** 2025-01-06

**Authors:** Dong Hye Suh, Tae‐Eun Kim, Sang Jun Lee, Su Jin Jeong, Eunsun Baek, Min Kyung Shin

**Affiliations:** ^1^ Dermatology Arumdaun Nara Dermatologic Clinic Seoul Korea; ^2^ Department of Dermatology, School of Medicine Kyung Hee University Hospital Seoul Korea; ^3^ Statistics Support Part Medical Science Research Institute, Kyung Hee University Seoul Korea; ^4^ Medical Affairs, Loreal Dermatological Beauty L'Oréal Korea Ltd. Seoul Korea

**Keywords:** acne vulgaris, adverse events, cheek acne, dermocosmetics, facial areas, patient adherence, quality of life, retinoids, skin sensitivity

## Abstract

**Background:**

Topical retinoids, while renowned for their efficacy in treating acne vulgaris, are often hampered by inter‐individual variability in tolerability. This challenge, primarily driven by side effects like erythema, scaling, and dryness, significantly impacts patient adherence and, ultimately, treatment outcomes.

**Aims:**

This prospective, multi‐center, observational study investigated the novel role of a specific dermocosmetic regimen as adjunctive therapy, focusing on its ability to mitigate retinoid‐induced side effects and enhance the overall tolerability of acne treatment regimens in a Korean population.

**Patients and Methods:**

We enrolled 304 patients receiving conventional acne therapies and integrated a standardized dermocosmetic regimen (foaming facial wash and moisturizer) for 12 weeks. Our primary endpoint assessed changes in skin sensitivity scores in both retinoid and non‐retinoid users. We incorporated a patient‐reported outcome measure evaluating acne's impact on quality of life across different facial areas as a secondary endpoint.

**Results:**

Our results revealed a significant improvement in skin sensitivity across both patient groups, effectively mitigating the anticipated heightened sensitivity in retinoid users. This finding suggests that dermocosmetics may hold the key to unlocking consistent, age‐independent tolerance to retinoid therapy. Furthermore, we observed a compelling correlation between improvements in cheek acne and enhanced quality of life, highlighting the profound psychological impact of this specific facial area.

**Conclusions:**

This study pioneers a new understanding of holistic acne management, emphasizing the synergistic potential of dermocosmetics in enhancing treatment adherence, improving long‐term outcomes, and ultimately transforming patients' lives.

## 1 Introduction

While clinically characterized by inflammatory lesions, acne vulgaris carries a significant psychosocial impact that extends beyond its dermatological manifestations, particularly in the domains of emotions, daily activities, social interactions, work/study, and interpersonal relationships [1]. This impact on quality of life (QoL) is substantial, with studies indicating that acne can be as debilitating as severe chronic illnesses like asthma, epilepsy, and back pain [2, 3]. Therefore, effective acne management strategies should not only target clinical improvement but also prioritize QoL improvements. One crucial aspect of this is mitigating the adverse effects of acne treatment, particularly the dryness and irritation commonly associated with retinoids, which can lead to treatment discontinuation and ultimately hinder clinical success [4]. Integrating dermocosmetics that address dryness and irritation alongside retinoid therapy may offer a multifaceted approach to improve patient adherence and ultimately enhance their quality of life by enabling consistent treatment and better clinical outcomes.

Retinoids are considered a cornerstone of acne management due to their ability to target multiple pathogenic factors, including comedolytic, anti‐inflammatory, and normalizing effects on follicular keratinization. Retinoids effectively address abnormal desquamation and increased sebum production, key factors in the development of microcomedones, the precursors to acne lesions. They achieve this by reducing keratinocyte proliferation, promoting differentiation, and blocking inflammatory pathways. However, despite their efficacy, the use of retinoids is sometimes hampered by inter‐individual variability in tolerability. Side effects, primarily erythema, scaling, dryness, and burning, can occur, particularly in the initial weeks of treatment, potentially impacting patient adherence and treatment success [5].

Systemic and topical retinoids, while highly effective, are known to induce skin sensitivity in a significant proportion of patients, posing a challenge to treatment adherence and therapeutic success [6]. This challenge has prompted the exploration of adjunctive therapies, such as dermocosmetics, to mitigate these side effects. Recent findings indicate that specific dermocosmetic regimens may hold promise in enhancing the tolerability of retinoid‐based acne treatments. By incorporating ingredients designed to fortify the skin barrier, reduce inflammation, and support a healthy skin microbiome, dermocosmetics may provide a synergistic approach to improving patient comfort and optimizing treatment outcomes [7, 8].

This prospective, multi‐center, observational study aimed to investigate the novel role of a specific dermocosmetic regimen as adjunctive therapy in mitigating retinoid‐induced side effects and enhancing the overall tolerability of acne treatment regimens in a Korean population. In addition to evaluating the impact of the dermocosmetic regimen on treatment‐induced skin sensitivity across different age groups and treatment modalities, this study sought to explore a novel aspect of acne's impact by investigating the relationship between acne location and quality of life. We hypothesized that the location of acne lesions could differentially affect patients' quality of life, even within the relatively small area of the face. This analysis aimed to identify whether the regimen demonstrated particular efficacy in improving acne and its associated quality of life impact in specific facial regions. Furthermore, recognizing the intricate relationship between the physical and psychological burden of acne, we embarked on an unprecedented exploration of the impact of acne severity in specific facial areas on patients' quality of life.

## 2 Materials and Methods

This research was designed as a multi‐center, prospective, observational study to comprehensively assess the real‐world efficacy and tolerability of a dermocosmetic regimen when employed as adjunctive therapy in the management of patients with acne vulgaris across a spectrum of disease severity. The study was strategically conducted across 13 dermatology clinics strategically distributed throughout Seoul, South Korea. This multi‐center approach was chosen to ensure the recruitment of a diverse patient population that accurately reflects real‐world clinical practice and enhances the generalizability of the study's findings.

Patient recruitment was conducted from June 2023 to November 2023, ultimately enrolling 304 participants who met the pre‐defined inclusion criteria. To be eligible for participation, patients had to be at least 14 years of age and present with facial acne. Patient facial acne severity was assessed and classified using the Global Acne Grading System [8], allowing for the evaluation of the adjunctive dermocosmetic regimen across a wide spectrum of disease severity.

A defining characteristic of this study was its focus on evaluating the dermocosmetic regimen within the context of ongoing conventional acne treatment, reflecting real‐world clinical scenarios. Therefore, all enrolled patients, irrespective of the specific agents being used, were required to be actively undergoing treatment with at least one conventional acne therapy at the time of study enrollment. Eligible treatment regimens included monotherapy with topical agents such as benzoyl peroxide (BPO), tretinoin, adapalene, trifarotene, azelaic acid, Adapalene 0.1%/BPO, Adapalene 0.3%/BPO, tretinoin/clindamycin, or BPO/clindamycin. Additionally, patients receiving a combination of topical treatments and systemic therapy with oral antibiotics were eligible for inclusion, as were those currently undergoing treatment with oral isotretinoin. Patients were excluded from participation if they were pregnant, breastfeeding, or under 14 years of age.

The presently investigated dermocosmetic (DC) regimen consists of a cleanser and a cream formulation that have been developed to help limit topical retinoid‐induced skin dryness (RISD) in subjects with acne. The DC cleanser (Effaclar H Iso Biome cream wash, La Roche‐Posay Laboratoire Dermatologique France) contains *Bixa orellana* seed extract, niacinamide 2%, mannose, and APF (Aqua Posae Filiformis). The DC cream (Effaclar H Iso Biome cream, La Roche‐Posay Laboratoire Dermatologique France) contains *Bixa orellana* seed extract, a plant extract that, in yet unpublished work, reduced sebum production, hyperkeratinization, and lipase activity from C. acnes. Furthermore, the cream is formulated with 2% niacinamide, panthenol, and the pre‐ andpost‐biotic APF. Niacinamide has been recognized for its ability to reduce sebum outflow, while mannose is known to enhance the biomechanical properties of the skin [9, 10]. APF reduces inflammation and helps restore the natural skin barrier [11, 12], while panthenol moisturizes the skin, thus further helping to restore the natural skin barrier [13].

The study was conducted over a 12‐week period. Patients were instructed to use the DC cleanser twice daily, in the morning and evening, during their regular facial cleansing routine. They were also instructed to apply the DC cream every evening after their prescribed acne medication. Alternatively, patients could choose to apply the DC cream every morning and their prescribed acne medication every evening. The amount of product used was left to the discretion of each patient.

At the initial visit, each participant underwent a comprehensive dermatological examination conducted by a board‐certified dermatologist. The dermatologist documented the patient's existing acne treatment regimen, including the specific medication(s), dosage, frequency of application, and duration of use. Additionally, acne characteristics were assessed, including the type (classified as comedonal, inflammatory, mixed, or nodulocystic based on the predominant lesion morphology), location (noting involvement of the facial T‐zone, hairline, cheeks, mandibular area, and/or trunk), and the presence or absence of any complications such as atrophic scars, post‐inflammatory erythema, and post‐inflammatory hyperpigmentation. The clinician also evaluated and graded the patient's sebum production on a scale of 0 (absence of seborrhea) to 10 (high seborrhea) and assessed erythema, desquamation, and dryness using a standardized 4‐point scale (0 = absent, 1 = mild, 2 = moderate, and 3 = severe) to determine objective skin sensitivity.

This study investigates the efficacy and tolerability of a two‐product dermocosmetic regimen in mitigating topical RISD in patients undergoing acne treatment. This study aims to evaluate the potential of this adjunctive dermocosmetic approach to improve patient comfort and adherence to treatment.

A total of 304 patients were enrolled in this study and categorized into two groups based on their prescribed acne medication: a retinoid group (*n* = 157) and a non‐retinoid group (*n* = 147). Both groups received the same dermocosmetic regimen as an adjunctive therapy for a period of 12 weeks. The primary endpoint of this study was the change in investigator‐assessed skin sensitivity scores from baseline to Week 12, evaluated separately for the retinoid and non‐retinoid cohorts. Skin sensitivity, encompassing erythema, desquamation, and dryness, was assessed using a 4‐point scale (1 = absent, 2 = mild, 3 = moderate, and 4 = severe) for each parameter, with a cumulative sum score also calculated.

This comparative approach aimed to elucidate the potential of the dermocosmetic regimen to mitigate the well‐documented skin sensitivity associated with topical retinoid use, a common challenge encountered in the management of acne [14]. In addition to the investigator's assessment, patients were also asked to rate their own skin sensitivity across several parameters, including itching, tingling sensation, burning sensation, and painful sensation. These were also evaluated using a 4‐point scale (1 = absent, 2 = mild, 3 = moderate, and 4 = severe) for each parameter. This two‐pronged approach, incorporating both objective investigator assessments and subjective patient‐reported outcomes, aimed to provide a comprehensive evaluation of the dermocosmetic regimen's impact on skin sensitivity. This multifaceted perspective is critical in addressing the multifaceted nature of RISD, which encompasses both clinically observable signs and individually experienced symptoms.

Next, we incorporated a stratified analysis of both investigator‐assessed and patient‐reported skin sensitivity scores as secondary endpoints. Participants were categorized into age groups within both the retinoid and non‐retinoid treatment cohorts, allowing us to assess whether the regimen's ability to mitigate dryness and irritation differed based on age and concurrent acne treatment.

Also, we included, as a final endpoint analysis, an assessment of acne's impact on quality of life across four distinct facial regions: the T‐zone, hairline (referred to as the “hair zone”), cheeks, and chin. This approach aimed to provide a more nuanced understanding of the psychosocial burden of acne, recognizing that even within a single individual, the location of lesions could significantly influence their perception of self and their interactions with others. Furthermore, to evaluate the effectiveness of the dermocosmetic regimen in improving quality of life across these different facial areas, we analyzed changes in patient‐reported outcomes over the 3‐month study period. This analysis aimed to identify if the regimen demonstrated particular efficacy in improving acne and its associated quality‐of‐life impact in specific facial regions.

All statistical analyses were conducted using SAS 9.4 software (SAS Institute, Cary, NC, USA). Descriptive statistics were used to characterize the study variables. For quantitative variables, such as skin sensitivity scores, means, standard deviations, medians, and interquartile ranges were calculated. Qualitative variables, such as treatment group allocation (retinoid vs. non‐retinoid), were summarized using frequencies and percentages. To assess the primary endpoint, which involved comparing proportions of patients experiencing changes in skin sensitivity, a Z‐test for comparison of proportions was used. Changes in skin sensitivity scores between baseline and Week 12 were evaluated using the Wilcoxon signed‐rank test, a non‐parametric test suitable for paired data that may not follow a normal distribution.

Patient satisfaction and tolerance were assessed using a binary approach, categorizing responses as either “favorable” or “unfavorable.” A proportion comparison test was then performed to compare these proportions between groups or across time points. For all analyses, a significance level of *p* < 0.05 was considered statistically significant.

## 3 Results

### 3.1 Study Population and Baseline Characteristics

**FIGURE 1:**
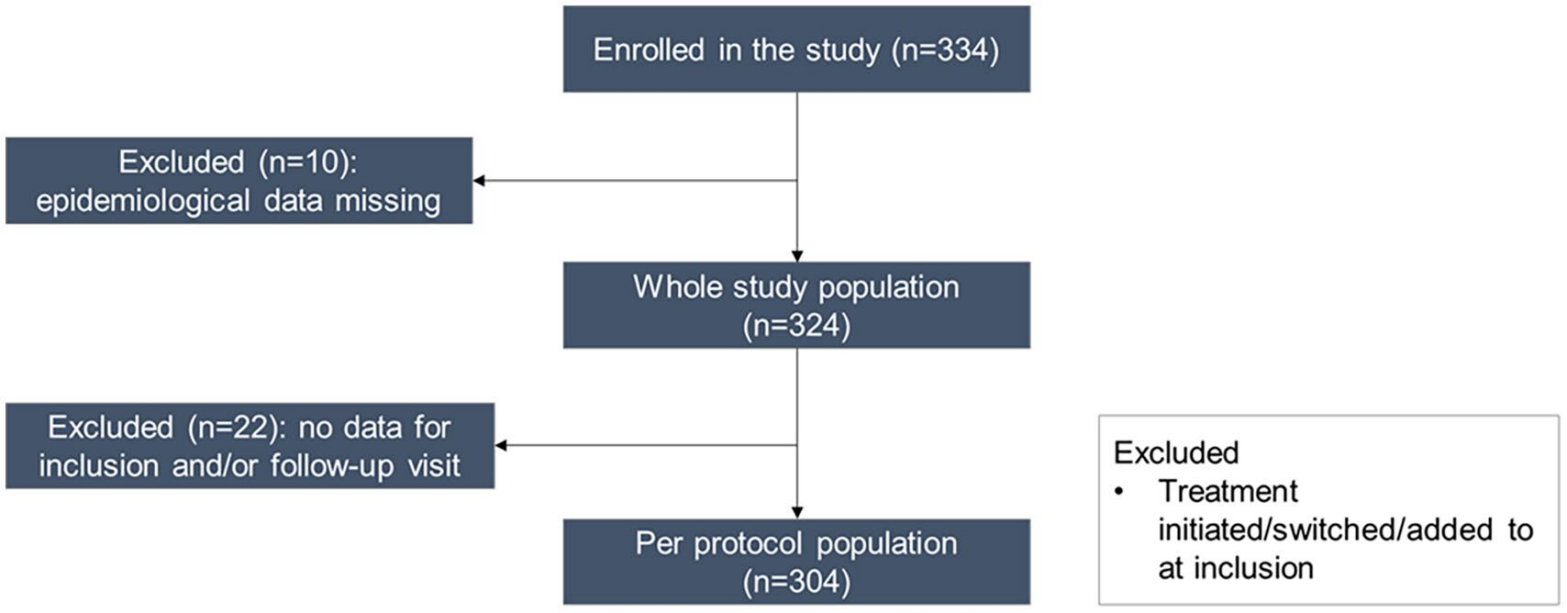
Participant flow through the study.

A total of 304 participants completed the 12‐week study, representing a per‐protocol population derived from an initial enrollment of 334 individuals (Figure 1). Ten participants were excluded due to missing epidemiological data, and an additional 22 were excluded due to the absence of data for either the inclusion or follow‐up visits. This rigorous selection ensured a robust dataset for analysis, minimizing potential bias from missing data points.

Among the 304 participants included in the final analysis, baseline demographic and clinical characteristics are presented in Table 1. The mean age of the participants was 26.4 ± 7.5 years, with a slightly higher representation of females (55.9%). Baseline characteristics were largely comparable between the retinoid and non‐retinoid treatment groups, indicating a balanced distribution of potential confounding factors. However, as expected, a significantly higher proportion of patients in the retinoid group presented with severe acne at baseline, reflecting the clinical use of retinoids in managing more challenging cases.

**TABLE 1:** Baseline characteristics of the study population.

	Total (*n* = 304)	Retinoid (*n* = 157)	Non‐retinoid (*n* = 147)	p*
Sex				
Male	134 (44.08%)	70 (44.59%)	64 (43.54%)	0.8540
Female	170 (55.92%)	87 (55.41%)	83 (56.46%)	
Age group (years)				
19 years and under	66 (21.71%)	30 (19.11%)	36 (24.49%)	0.4389
20–34 years	189 (62.17%)	99 (63.06%)	90 (61.22%)	
Over 35 years	49 (16.12%)	28 (17.83%)	21 (14.29%)	
Phototype				
II	25 (8.22%)	10 (6.370%)	15 (10.20%)	0.2698
III	147 (48.36%)	73 (46.50%)	74 (50.34%)	
IV	132 (43.42%)	74 (47.13%)	58 (39.46%)	
Acne onset (years)				
5 years and under	240 (78.95%)	123 (78.34%)	117 (79.59%)	0.7897
Over 5 years	64 (21.05%)	34 (21.66%)	30 (20.41%)	
Acne type				
Comedonal	29 (9.54%)	13 (8.28%)	16 (10.88%)	0.0008
Inflammatory	100 (32.89%)	37 (23.57%)	63 (42.86%)	
Mixed	173 (56.91%)	105 (66.88%)	68 (46.26%)	
Nodulocystic	2 (0.66%)	2 (1.27%)	0 (0.00%)	
Prescription modality				
Topical alone	111 (36.51%)	59 (37.58%)	52 (35.37%)	< 0.0001
Topical + systemic	124 (40.79%)	29 (18.47%)	95 (64.63%)	
Systemic treatment	69 (22.70%)	69 (43.95%)	0 (0.00%)	

*Note: N* (%), *chi‐square test.

**TABLE 2:** Baseline characteristics of patients by presence of acne in each facial area.

	19 years and under (*n* = 66)	20–34 years (*n* = 189)	Over 35 years (*n* = 49)	p*
Sex				
Male	35 (53.03%)	84 (44.44%)	15 (30.61%)	0.0561
Female	31 (46.97%)	105 (55.56%)	34 (69.39%)	
Common acne location				
T‐zone	56 (84.85%)	126 (66.67%)	16 (32.65%)	< 0.0001
Hairline	44 (66.67%)	87 (46.03%)	8 (16.33%)	< 0.0001
Cheek	59 (89.39%)	159 (84.13%)	31 (63.27%)	0.0007
Jawline	28 (42.42%)	108 (57.14%)	42 (85.71%)	< 0.0001
Acne type				
Mixed	46 (69.70%)	111 (58.73%)	16 (32.65%)	
Non‐mixed	20 (30.30%)	78 (41.27%)	33 (67.35%)	0.0003
Retinoid				
Retinoid	30 (45.45%)	99 (52.38%)	28 (57.14%)	0.4389
Non‐retinoid	51 (77.27%)	126 (66.67%)	27 (55.10%)	0.0427

*Note: N*(%), # = comedonal/2 = inflammatory/4 = nodulocystic, *chi‐square test.

Further analysis of baseline characteristics revealed no significant differences in the distribution of sex and age groups between the retinoid and non‐retinoid groups (Table 2). However, there was a statistically significant difference in the types of acne observed. Patients in the retinoid group were more likely to present with mixed acne (66.9%) compared to the non‐retinoid group (46.3%), while the non‐retinoid group had a higher proportion of patients with inflammatory acne (42.9% vs. 23.6%, *p* = 0.0010). This finding aligns with the clinical practice of reserving retinoids for more severe or treatment‐resistant acne.

Prescribed treatment modalities differed significantly between the groups. All patients in the retinoid group (100%) received a combination of topical retinoids and oral isotretinoin, whereas no patients in the non‐retinoid group received this combination. Conversely, the non‐retinoid group had a higher proportion of patients receiving either topical therapy alone (35.4% vs. 37.6%) or a combination of topical and systemic therapies excluding oral isotretinoin (64.6% vs. 18.5%). This difference highlights the distinct treatment approaches for acne, tailored to its severity and individual patient characteristics.

Prior to the initiation of the adjunctive dermocosmetic regimen, a comprehensive analysis of demographic and clinical characteristics was conducted, focusing on the location of acne lesions. As demonstrated in Table 1, the majority of participants were female (55.92%), with the 20‐ to 34‐year age group being the most represented (62.17%). The majority had developed acne within the preceding 5 years (78.95%), and the mixed acne type was most prevalent (56.91%). Post‐inflammatory erythema was identified as the most frequent acne complication, observed in 76.26% of participants. A significant portion of patients (40.79%) were already using a combination of topical and systemic treatments at the study's commencement, with topical retinoids being the most commonly prescribed topical medication (29.29%).

It is crucial to highlight that acne lesion distribution was frequently not limited to a single anatomical location. Participants often presented with acne affecting multiple facial areas concurrently, such as the T‐zone and hairline, or the cheeks and jawline. This observation underscores the intricate nature of acne distribution and emphasizes the necessity for comprehensive treatment approaches that effectively target all affected areas.

### 3.2 Assessment of Treatment‐Induced Skin Sensitivity

**FIGURE 2:**
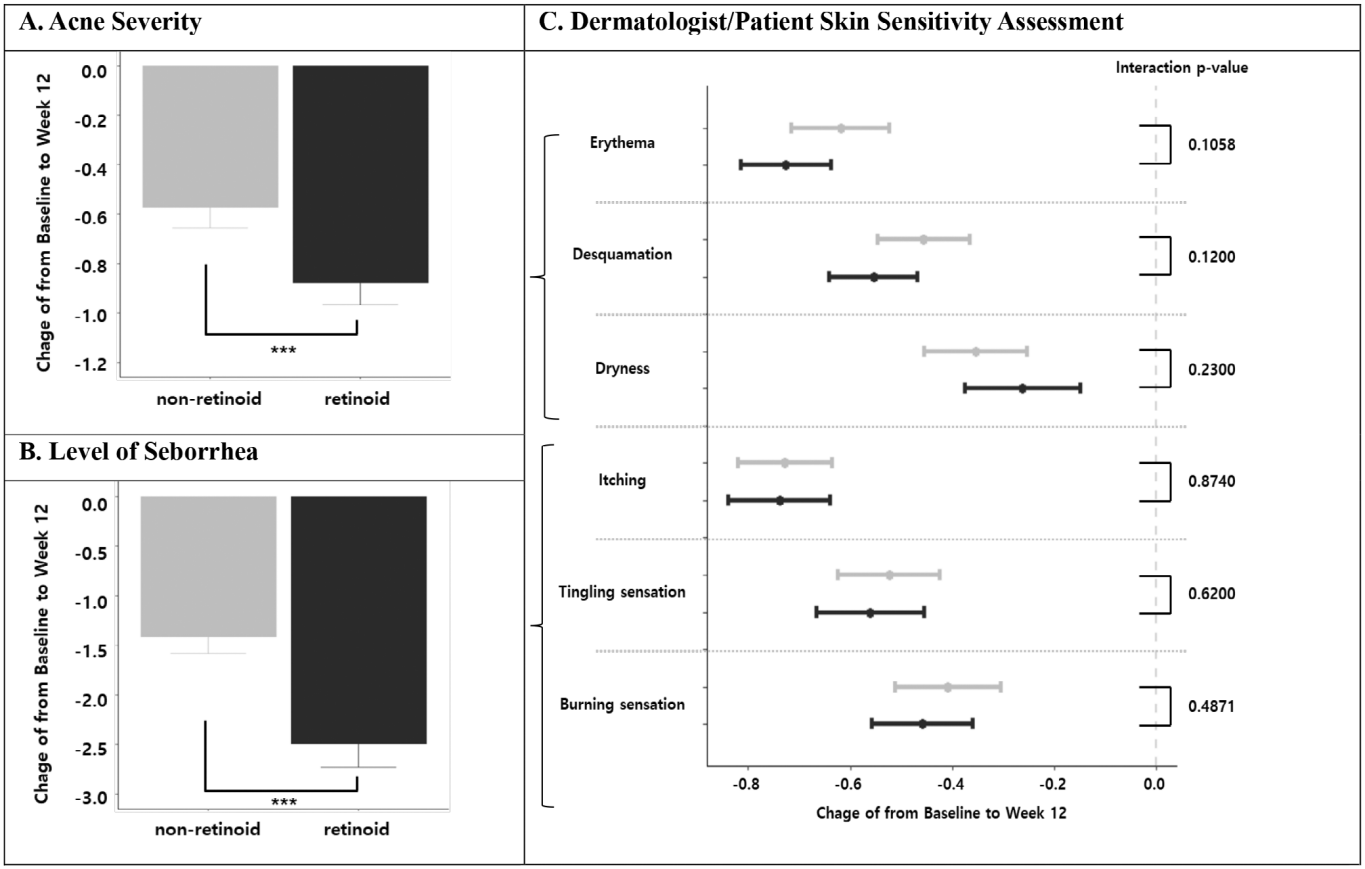
Changes in investigator‐assessed and patient‐reported skin sensitivity scores from baseline to Week 12. (A) Changes in acne severity; (B) changes in sebum secretion; (C) changes in investigator‐assessed and patient‐reported skin sensitivity scores. The types of investigator‐assessed sensitivity scores are erythema, and desquamation. The types of patient‐reported skin sensitivity scores are itching, tingling, and burning sensation; *p‐value of interaction with the retinoid group is a result of a multiple mixed model analysis adjusted for sex, age, Fitzpatrick's phototype, acne type, and prescription modality.

The primary objective of this study was to evaluate the efficacy of the adjunctive dermocosmetic regimen in mitigating skin sensitivity associated with conventional acne treatment, focusing on both investigator‐observed signs and patient‐reported symptoms. To achieve this, we conducted a comparative analysis of changes in skin sensitivity scores from baseline to Week 12, stratified by treatment group (retinoid vs. non‐retinoid). Both the retinoid and non‐retinoid groups demonstrated significant reductions in investigator‐assessed skin sensitivity scores from baseline to Week 12 (Figure 2).

Though baseline patient‐reported scores for itching, tingling, burning, and their cumulative sum were comparable between groups, only the retinoid group exhibited statistically significant improvements after 12 weeks. This study compared the changes in acne severity, sebum secretion, and skin sensitivity scores between a non‐retinoid group and a retinoid group (Figure 2).

A significant reduction in acne severity was observed in both groups, with the retinoid group exhibiting a greater improvement compared to the non‐retinoid group (−0.88 [CI: −0.97, −0.79] vs. −0.57 [CI: −0.66, −0.49], respectively, Figure 2A). Similarly, sebum secretion significantly decreased in both groups, with a more pronounced reduction observed in the retinoid group (−2.49 [CI: −2.73, −2.25] vs. −1.41 [CI: −1.58, −1.25], respectively, Figure 2B).

Dermatologist‐assessed skin sensitivity, as measured by erythema, desquamation, and dryness scores, demonstrated reductions in both the non‐retinoid and retinoid groups. The non‐retinoid group showed score decreases of −0.62 (CI: −0.72, −0.52), −0.46 (CI: −0.55, −0.37), and −0.35 (CI: −0.45, −0.25) for erythema, desquamation, and dryness, respectively. Similarly, the retinoid group exhibited reductions of −0.73 (CI: −0.81, −0.64), −0.55 (CI: −0.64, −0.47), and − 0.26 (CI: −0.37, −0.15) for the same parameters. However, these changes were not statistically different between the groups.

Patient‐reported skin sensitivity, encompassing self‐reported symptoms of itching, tingling, and burning sensations, also demonstrated improvements in both groups. The non‐retinoid group reported reductions of −0.73 (CI: −0.82, −0.64) for itching, −0.52 (CI: −0.62, −0.42) for tingling, and − 0.41 (CI: −0.51, −0.30) for burning. The retinoid group reported similar changes: −0.74 (CI: −0.84, −0.64) for itching, −0.56 (CI: −0.67, −0.45) for tingling, and −0.46 (CI: −0.56, −0.36) for burning. Again, these changes were not statistically different between the two treatment groups.

Sub‐group analysis based on acne location (T‐zone, hairline, cheek, and jawline) revealed consistent findings, indicating no significant differences in sensitivity changes between the non‐retinoid and retinoid groups across various anatomical sites (Data S1).

### 3.3 Age‐Related Variations in Response to the Dermocosmetic Regimen

**FIGURE 3:**
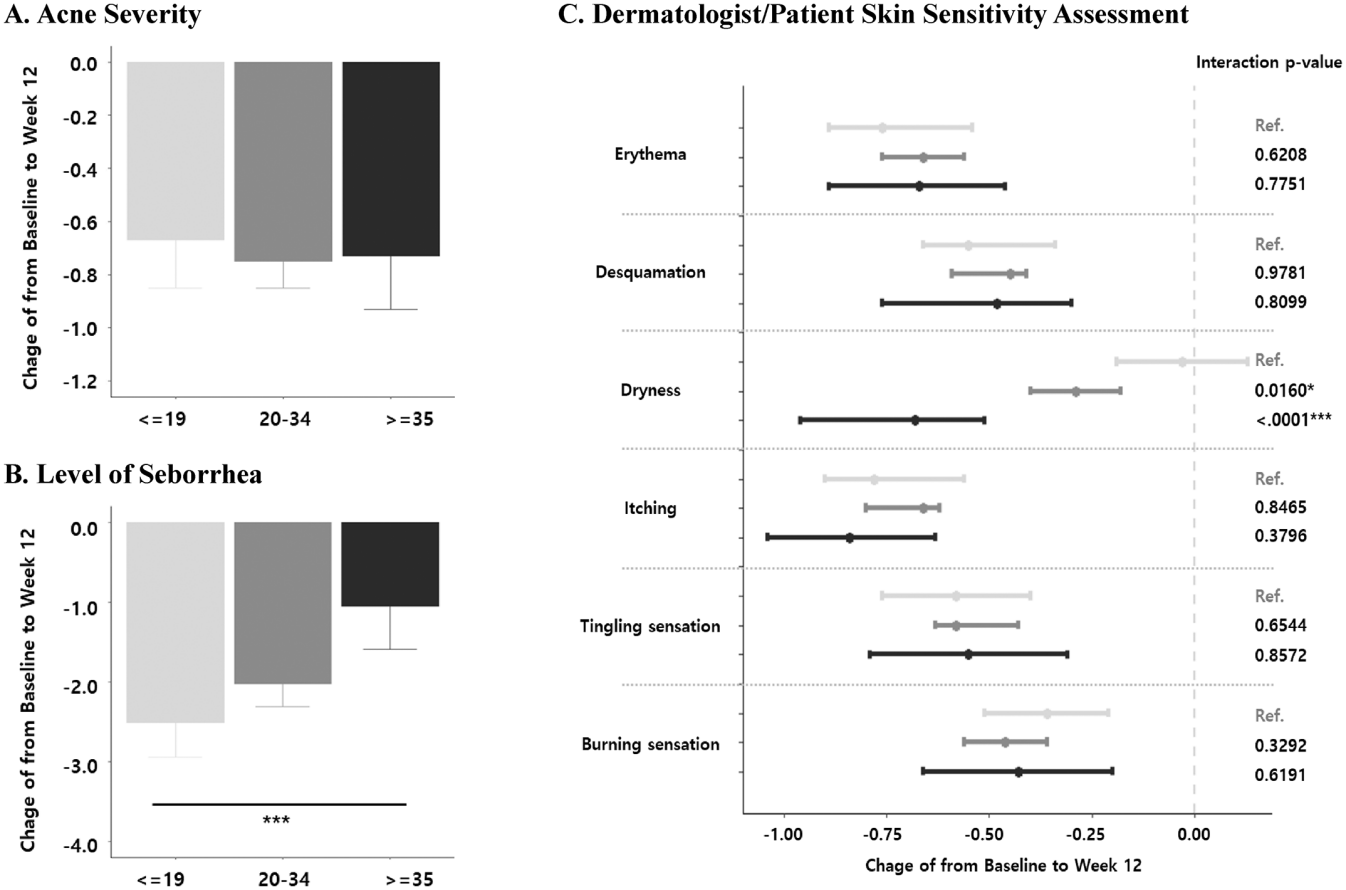
Changes in investigator‐assessed and patient‐reported skin sensitivity scores from baseline to Week 12, stratified by age group. (A) Changes in acne severity; (B) changes in sebum secretion; (C) changes in investigator‐assessed and patient‐reported skin sensitivity scores. The types of investigator‐assessed sensitivity scores are erythema, desquamation, and desquamation. The types of patient‐reported skin sensitivity scores are itching, tingling, and burning sensation. *p‐value of interaction with the retinoid group is a result of multiple mixed model analyses adjusted by sex, age, phototype, acne type, and prescription modality.

Recognizing the inherent age‐related changes in skin barrier function and sensitivity [7], we conducted a stratified analysis to evaluate whether the dermocosmetic regimen's efficacy in mitigating treatment‐induced skin sensitivity varied across different age groups. As expected, baseline skin sensitivity scores, both investigator‐assessed and patient‐reported, generally increased with age. Contrary to our initial hypothesis that older individuals might experience less pronounced improvement, our analysis revealed a consistent and significant reduction in both investigator‐assessed and patient‐reported skin sensitivity scores across all age groups in both the retinoid and non‐retinoid cohorts (Figure 3). These improvements were statistically significant (*p* < 0.05) for all age groups and treatment modalities, as demonstrated by the negative beta estimates and 95% confidence intervals that did not cross zero.

Changes in acne severity, sebum secretion, and skin sensitivity were also analyzed across different age groups (≤ 19, 20–34, ≥ 35 years) (Figure 3). Acne severity demonstrated significant improvement across all age groups. Reductions were observed for the ≤ 19 years group (−0.67 [CI: −0.85, −0.48]), 20–34 years group (−0.75 [CI: −0.85, −0.65]), and ≥ 35 years group (−0.73 [CI: −0.93, −0.54]) (Figure 3A). These changes in acne severity did not differ significantly between the age groups.

Sebum secretion also decreased across all age groups, but significant differences were observed in the magnitude of change (Figure 3B). The youngest age group (≤ 19 years) showed the greatest reduction in sebum secretion (−2.51 [CI: −2.94, −2.07]), followed by the 20–34 years group (−2.02 [CI: −2.31, −1.74]), and lastly the ≥ 35 years group (−1.05 [CI: −1.59, −0.51]). This difference was particularly pronounced between the ≤ 19 years and ≥ 35 years groups (*p* < 0.0001). Dermatologist‐assessed skin sensitivity scores for erythema and desquamation showed improvement across all age groups, without statistically significant differences between them. However, dryness scores significantly differed based on age (Figure 3C). While the ≤ 19 years group demonstrated a reduction of −0.03 (CI: −0.19, 0.13), the 20–34 years and ≥ 35 years groups showed greater improvements: −0.29 (CI: −0.40, −0.18) and −0.73 (CI: −0.96, −0.51), respectively. The difference in dryness score changes was significant between the ≤ 19 years group compared to both the 20–34 years group (*p*‐value: 0.0160) and the ≥ 35 years group (*p* < 0.0001). Patient‐reported skin sensitivity, encompassing self‐reported itching, tingling, and burning sensations, showed improvements in all age groups. However, these changes were not significantly different between the age groups (Figure 3C).

Similar trends were observed in sub‐group analyses stratified by acne location (T‐zone, hairline, cheek, and jawline), suggesting consistent results across different anatomical areas (Data S2).

### 3.4 Impact of Acne Location on Quality of Life

To investigate this, we included, as a final endpoint analysis, an assessment of acne's impact on quality of life across four distinct facial regions: the T‐zone, hairline (referred to as the “hair zone”), cheeks, and chin. This approach aimed to provide a more nuanced understanding of the psychosocial burden of acne, recognizing that even within a single individual, the location of lesions could significantly influence their perception of self and their interactions with others. Furthermore, to evaluate the effectiveness of the dermocosmetic regimen in improving quality of life across these different facial areas, we analyzed changes in patient‐reported outcomes over the 3‐month study period.

**FIGURE 4:**
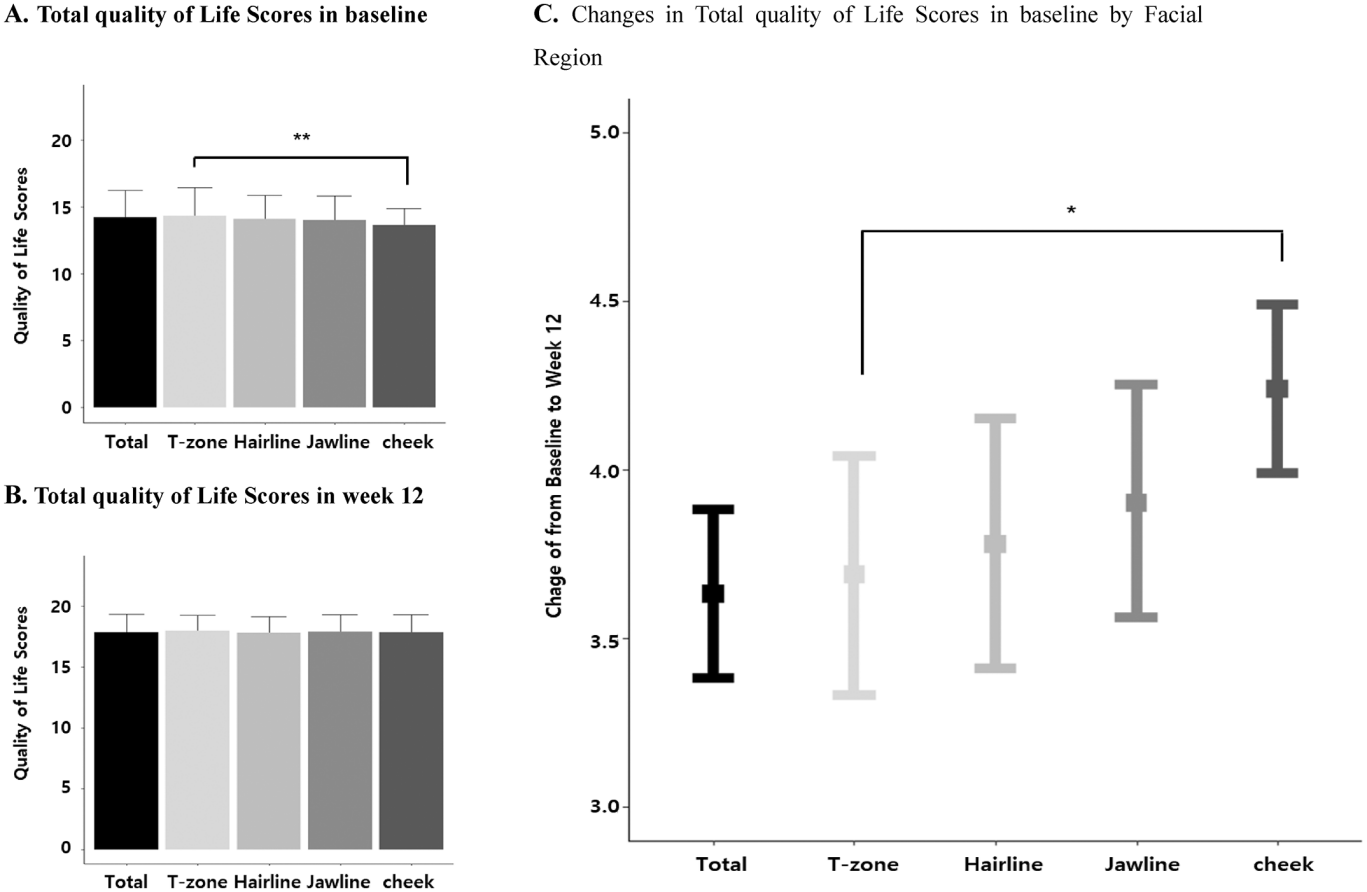
Change in quality of life scores from baseline to Week 12, stratified by facial region. (A) Total quality of life scores at baseline by facial region; (B) total quality of life scores at Week 12 by facial region; (C) changes in total quality of life scores from baseline by facial region. *p‐value of interaction with the facial region is a result of multiple mixed model analyses adjusted for sex, age, phototype, acne type, and prescription modality.

This study also evaluated the influence of acne location on quality‐of‐life (QoL) changes (Figure 4).

At baseline, the overall QoL, represented by the total QoL score, was 14.24 ± 1.99. Scores varied across different facial areas, with the T‐zone exhibiting the highest score (14.35 ± 2.09), followed by the hairline (14.11 ± 1.77), jawline (14.02 ± 1.82), and lastly the cheeks (13.65 ± 1.25) (Figure 4A). This difference in baseline QoL scores was statistically significant, with the cheeks demonstrating a notably lower score compared to the T‐zone.

Following cosmetic intervention, a significant improvement in the overall QoL was observed, with the total score increasing to 17.87 ± 1.48, reflecting a significant increase of 3.63 points (CI: 3.38, 3.88) (Figure 4B). This improvement was consistent across all facial areas, with each location showing a statistically significant increase in QoL scores: T‐zone (3.69 [CI: 3.33, 4.04]), hairline (3.78 [CI: 3.41, 4.15]), jawline (3.90 [CI: 3.56, 4.25]), and cheeks (4.24 [CI: 3.99, 4.49]) (Figure 4C). Notably, the most substantial improvement in QoL was observed in the cheek area, which also had the lowest baseline score, with a significantly greater increase compared to the T‐zone.

Further analysis of individual QoL sub‐scores (Q1‐Q5) confirmed these findings. All sub‐scores demonstrated significant improvements: Q1 (from 3.13 ± 0.69 to 3.92 ± 0.30), Q2 (from 2.78 ± 0.66 to 3.79 ± 0.41), Q3 (from 3.88 ± 0.36 to 3.97 ± 0.18), Q4 (from 2.26 ± 0.59 to 2.99 ± 0.47), and Q5 (from 2.18 ± 0.43 to 3.21 ± 0.70) (Data S2). Similar patterns were observed when analyzing changes in QoL sub‐scores across different age groups and acne locations, suggesting consistent results (Data S3).

Interestingly, despite significant improvements in acne severity and sebum secretion across all facial regions in both treatment groups (Figure 4), the impact of acne location on quality of life persisted after the 12‐week treatment period. While overall quality‐of‐life scores improved significantly in all areas, the most substantial improvements were observed in patients who experienced a reduction in cheek acne. Conversely, even if acne improved in other areas, persistent cheek acne continued to exert a disproportionately negative impact on quality of life.

This finding highlights the importance of considering acne location when assessing treatment efficacy and patient satisfaction. While overall improvement in acne severity is crucial, clinicians should pay particular attention to acne affecting the cheeks, as its resolution appears to have a greater impact on patient quality of life. This knowledge can inform treatment decisions, allowing clinicians to prioritize strategies that effectively target cheek acne and alleviate the associated psychosocial burden.

## 4 Discussion

This multi‐center, prospective, observational study aimed to evaluate the real‐world efficacy and tolerability of a two‐product dermocosmetic regimen as adjunctive therapy for patients with acne vulgaris undergoing conventional treatment. Our findings demonstrate that the regimen significantly reduced both investigator‐assessed and patient‐reported skin sensitivity across all age groups and treatment modalities, highlighting its potential to improve the tolerability and adherence to conventional acne treatments, particularly for those using retinoids. While baseline scores for erythema, desquamation, and dryness were slightly higher in the retinoid group, indicating a trend toward greater baseline sensitivity, both groups showed comparable and statistically significant reductions in all three parameters and the cumulative sum score. This suggests that the adjunctive dermocosmetic regimen effectively mitigated skin sensitivity induced by both retinoid and non‐retinoid acne treatments.

Interestingly, while investigator‐observed skin sensitivity showed significant improvement in both groups, patient‐reported sensitivity revealed a different pattern. This discrepancy between investigator and patient assessments highlights the subjective nature of skin sensitivity and underscores the importance of considering both objective signs and patient‐reported symptoms when evaluating treatment efficacy. These findings suggest that the adjunctive dermocosmetic regimen may be particularly beneficial for patients using retinoids, who are known to experience higher rates of skin sensitivity [7]. By mitigating both the clinically observable signs of skin sensitivity and the subjective discomfort experienced by patients, the dermocosmetic regimen could potentially improve treatment adherence and overall patient satisfaction in this population.

Contrary to our initial hypothesis, the dermocosmetic regimen proved effective in mitigating skin sensitivity in both retinoid and non‐retinoid users. This suggests a broader applicability of this adjunctive approach than initially anticipated. The observed improvement in skin barrier function, reflected in the reduction of objective signs such as erythema, desquamation, and dryness, likely contributed to the overall reduction in sensitivity. This finding underscores the importance of addressing skin barrier disruption as a crucial component of acne management, especially in the context of potentially irritating therapies.

Our stratified analysis by age revealed that the dermocosmetic regimen effectively mitigated treatment‐induced skin sensitivity regardless of age, demonstrating its potential benefit even in older individuals with intrinsically more sensitive skin due to age‐related physiological changes. Notably, all age groups experienced statistically significant improvements in both investigator‐assessed and patient‐reported skin sensitivity scores, further supporting the regimen's broad applicability. As described by Farage et al. [15], skin undergoes significant age‐related changes, impacting its barrier function and sensitivity. The stratum corneum, the outermost layer of the epidermis responsible for maintaining hydration and protection, becomes thinner and more fragile, which can lead to increased transepidermal water loss, resulting in dryness and a compromised skin barrier. Additionally, the production of ceramides, lipids that play a crucial role in maintaining skin hydration and barrier function, declines with age. These changes contribute to a heightened sensitivity to environmental aggressors and topical agents, making older individuals more susceptible to irritation and dryness.

Interestingly, acne severity and sebum secretion also showed significant improvements from baseline to Week 12 across all age groups. Both retinoid and non‐retinoid groups experienced a reduction in acne severity scores, although the magnitude of improvement was greater in the retinoid group, aligning with their typically more severe baseline acne. Similarly, sebum secretion scores decreased significantly in both groups and across all age ranges. This observation suggests that the dermocosmetic regimen, while not directly targeting acne, might contribute to a more favorable environment for acne resolution by improving skin barrier function and reducing inflammation.

The use of topical retinoids in acne treatment, while highly effective, can be complicated by their tendency to induce skin sensitivity. Even in younger individuals, retinoids are known to cause dryness and irritation, which may pose challenges to treatment adherence [16]. This sensitivity may be further compounded in older individuals, whose skin undergoes significant age‐related changes, including a thinner and more fragile stratum corneum, leading to a compromised skin barrier and heightened susceptibility to irritation [15]. This finding suggests that the dermocosmetic regimen effectively mitigated treatment‐induced skin sensitivity, regardless of age. Importantly, even in individuals with intrinsically more sensitive skin due to age‐related changes, the regimen significantly reduced both objective signs and subjective symptoms of irritation. Therefore, understanding the potential age‐related variations in response to the dermocosmetic regimen is crucial for optimizing its use.

In addition to evaluating the overall impact of the dermocosmetic regimen on skin sensitivity across different age groups, this study also sought to address a crucial yet often overlooked aspect of acne treatment: the impact of lesion location on patients' quality of life. While previous studies have primarily focused on the overall severity and improvement of acne [17], this study sought to investigate the impact of acne on quality of life in relation to specific facial areas.

This targeted assessment of the quality of life across different facial areas has significant clinical implications. While previous studies have primarily focused on the overall severity and improvement of acne [17], we hypothesized that the location of acne lesions could differentially affect patients' quality of life, even within the relatively small area of the face. We found that cheek acne had a significantly greater negative impact on patient‐reported quality of life compared to acne in other facial regions. This finding emphasizes the importance of considering acne location when assessing treatment efficacy and patient satisfaction.

By identifying specific regions where acne exerts the most significant impact, clinicians can tailor treatment strategies to address not only the clinical manifestations of acne but also the psychosocial concerns of individual patients. For example, a patient experiencing significant emotional distress due to persistent acne in the cheek area might benefit from a more aggressive treatment approach in that region, while a patient with milder acne primarily localized to the hairline might prioritize treatments that minimize the risk of scarring. This knowledge can inform treatment decisions, allowing clinicians to prioritize strategies that effectively target cheek acne and alleviate the associated psychosocial burden. For instance, when faced with a patient presenting with both cheek and forehead acne, a clinician might prioritize treatments that are known to be particularly effective for cheek lesions, even if those treatments are less effective for forehead acne. Similarly, a clinician might consider prescribing a combination therapy approach, using different modalities to target different facial regions based on their individual needs.

While this study provides valuable insights into the potential benefits of the dermocosmetic regimen, it is important to acknowledge its limitations. As an observational study, it cannot establish a causal relationship between the dermocosmetic regimen and the observed improvements in skin sensitivity and quality of life. Additionally, the reliance on subjective patient‐reported outcomes for assessing quality of life introduces potential for bias. Furthermore, this study underscores the importance of patient education and communication. Clinicians should openly discuss the potential impact of acne location on quality of life with their patients, emphasizing that their concerns are valid and that targeted treatment options are available. By fostering a collaborative and empathetic approach to treatment, clinicians can empower patients to actively participate in their care and ultimately achieve better outcomes, both clinically and psychosocially. Further research, including randomized controlled trials with larger sample sizes and longer follow‐up periods, is warranted to confirm these findings and further explore the regimen's long‐term effects.

## 5 Conclusion

This study suggests that the adjunctive use of the investigated dermocosmetic regimen effectively mitigates treatment‐induced skin sensitivity across a diverse range of acne patients, regardless of age or concurrent acne therapies. Notably, the regimen demonstrated a significant impact on improving quality of life, particularly in individuals with cheek acne, highlighting the importance of considering acne location in treatment planning. While further research is needed to confirm these findings, our results suggest that this dermocosmetic regimen may serve as a valuable adjunctive tool for improving patient adherence and overall treatment outcomes in the management of acne vulgaris.

## Author Contributions

D.S., S.L., and E.B. performed the research. D.S., T.K., and E.B. designed the research study. D.S., T.K., and M.S. conceived and designed the analysis. S.J. analyzed the data. D.S., T.K., E.B., and M.S. wrote the paper.

## Conflicts of Interest

The authors declare no conflicts of interest.

## Supporting information


Data S1.

## Data Availability

The data that support the findings of this study are available on request from the corresponding author. The data are not publicly available due to privacy or ethical restrictions.

## References

[1] N. Hazarika and M. Archana , “The Psychosocial Impact of Acne Vulgaris,” Indian Journal of Dermatology 61, no. 5 (2016): 515–520, 10.4103/0019-5154.190102. 27688440 PMC5029236

[2] A. O. Akinboro , O. I. Ezejiofor , F. O. Olanrewaju , et al., “The Impact of Acne and Facial Post‐Inflammatory Hyperpigmentation on Quality of Life and Self‐Esteem of Newly Admitted Nigerian Undergraduates,” Clinical, Cosmetic and Investigational Dermatology 11 (2018): 245–252, 10.2147/CCID.S158129. 29785134 PMC5955012

[3] S. Aktan , E. Ozmen , and B. Sanli , “Anxiety, Depression, and Nature of Acne Vulgaris in Adolescents,” International Journal of Dermatology 39, no. 5 (2000): 354–357, 10.1046/j.1365-4362.2000.00907.x. 10849125

[4] A. C. Narsa , C. Suhandi , J. Afidika , S. Ghaliya , K. M. Elamin , and N. Wathoni , “A Comprehensive Review of the Strategies to Reduce Retinoid‐Induced Skin Irritation in Topical Formulation,” Dermatology Research and Practice 2024 (2024): 5551774, 10.1155/2024/5551774. 39184919 PMC11344648

[5] J. Leyden , L. Stein‐Gold , and J. Weiss , “Why Topical Retinoids Are Mainstay of Therapy for Acne,” Dermatology and Therapy 7, no. 3 (2017): 293–304, 10.1007/s13555-017-0185-2. 28585191 PMC5574737

[6] V. Garofalo , M. V. Cannizzaro , S. Mazzilli , L. Bianchi , and E. Campione , “Clinical Evidence on the Efficacy and Tolerability of a Topical Medical Device Containing Benzoylperoxide 4%, Retinol 0.5%, Mandelic Acid 1% and Lactobionic Acid 1% in the Treatment of Mild Facial Acne: An Open Label Pilot Study,” Clinical, Cosmetic and Investigational Dermatology 12 (2019): 363–369, 10.2147/CCID.S182317. 31190944 PMC6526677

[7] A. Khammari , D. Kerob , A. L. Demessant , M. Nioré , and B. Dréno , “A Dermocosmetic Regimen Is Able to Mitigate Skin Sensitivity Induced by a Retinoid‐Based Fixed Combination Treatment for Acne: Results of a Randomized Clinical Trial,” Journal of Cosmetic Dermatology 23, no. 4 (2024): 1313–1319, 10.1111/jocd.16120. 38102855

[8] I. H. Bae , J. H. Kwak , C. H. Na , M. S. Kim , B. S. Shin , and H. Choi , “A Comprehensive Review of the Acne Grading Scale in 2023,” Annals of Dermatology 36, no. 2 (2024): 65–73, 10.5021/ad.23.094. 38576244 PMC10995619

[9] Z. D. Draelos , A. Matsubara , and K. Smiles , “The Effect of 2% Niacinamide on Facial Sebum Production,” Journal of Cosmetic and Laser Therapy 8, no. 2 (2006): 96–101, 10.1080/14764170600717704. 16766489

[10] M. Meunier , E. Chapuis , L. Lapierre , et al., “Mannose‐6‐Phosphate Complex and Improvement in Biomechanical Properties of the Skin,” Journal of Cosmetic Dermatology 20, no. 6 (2021): 1598–1610, 10.1111/jocd.14000. 33580613 PMC8251629

[11] Y. F. Mahe , M. J. Perez , C. Tacheau , et al., “A New *Vitreoscilla filiformis* Extract Grown on Spa Water‐Enriched Medium Activates Endogenous Cutaneous Antioxidant and Antimicrobial Defenses Through a Potential Toll‐Like Receptor 2/Protein Kinase C, Zeta Transduction Pathway,” Clinical, Cosmetic and Investigational Dermatology 6 (2013): 191–196, 10.2147/ccid.S47324. 24039440 PMC3770492

[12] H. Baldwin , C. Aguh , A. Andriessen , et al., “Atopic Dermatitis and the Role of the Skin Microbiome in Choosing Prevention, Treatment, and Maintenance Options,” Journal of Drugs in Dermatology 19, no. 10 (2020): 935–940, 10.36849/JDD.2020.5393. 33026777

[13] X. Zhang , D. Kerob , Z. Zhang , et al., “Efficacy and Safety of a Cream Containing Panthenol, Prebiotics, and Probiotic Lysate for Improving Sensitive Skin Symptoms,” Skin Research and Technology 30, no. 1 (2024): e13540, 10.1111/srt.13540. 38186043 PMC10772476

[14] E. Araviiskaia , J. L. Lopez Estebaranz , and C. Pincelli , “Dermocosmetics: Beneficial Adjuncts in the Treatment of Acne Vulgaris,” Journal of Dermatological Treatment 32, no. 1 (2021): 3–10, 10.1080/09546634.2019.1628173. 31211609

[15] M. A. Farage , K. W. Miller , P. Elsner , and H. I. Maibach , “Characteristics of the Aging Skin,” Advances in Wound Care 2, no. 1 (2013): 5–10, 10.1089/wound.2011.0356. 24527317 PMC3840548

[16] S. Jaiswal , S. Jawade , B. Madke , and S. Gupta , “Recent Trends in the Management of Acne Vulgaris: A Review Focusing on Clinical Studies in the Last Decade,” Cureus 16, no. 3 (2024): e56596, 10.7759/cureus.56596. 38646359 PMC11031619

[17] J. Tan , S. Beissert , F. Cook‐Bolden , et al., “Impact of Facial and Truncal Acne on Quality of Life: A Multi‐Country Population‐Based Survey,” JAAD International 3 (2021): 102–110, 10.1016/j.jdin.2021.03.002. 34409378 PMC8362284

