# Supplementary material for: Enhanced Tolerability and Improved Outcomes in Acne Management: A Real‐World Study of Dermocosmetic Adjunctive Therapy
Source: J Cosmet Dermatol. 2025 Jan 6;24(1):e16772. doi: 10.1111/jocd.16772 (PMC11701793; doi:10.1111/jocd.16772)
Supplement: Supplementary file 1 — Data S1. [file JOCD-24-e16772-s001.docx]

**Supplementary 1.** Changes in Acne Severity Scores and Sebum Secretion Scores from Baseline to Week 12, Stratified by Facial Region

| **variable** | **location** | **retinoid** | | | | **non-retinoid** | | | | **interaction of  p-value** |
| --- | --- | --- | --- | --- | --- | --- | --- | --- | --- | --- |
|  |  | **Baseline** | **Week 12** | **Difference** | **paired p-value** | **Baseline** | **Week 12** | **Difference** | **paired p-value** |  |
| **Acne severity** | **Total** | 2.55 ± 0.65 | 1.68±0.53 | -0.88±0.56 | <.0001 | 2.30±0.54 | 1.73±0.50 | -0.57±0.52 | <.0001 | <.0001 |
|  | **T-zone** | 2.64±0.67 | 1.74±0.54 | -0.90±0.57 | <.0001 | 2.32±0.55 | 1.77±0.47 | -0.55±0.54 | <.0001 | 0.0010 |
|  | **Hairline** | 2.72±0.70 | 1.78±0.54 | -0.94±0.54 | <.0001 | 2.34±0.56 | 1.79±0.45 | -0.56±0.53 | <.0001 | 0.0029 |
|  | **Cheek** | 2.66±0.65 | 1.76±0.52 | -0.91±0.57 | <.0001 | 2.35±0.56 | 1.77±0.49 | -0.58±0.53 | <.0001 | 0.0006 |
|  | **Jawline** | 2.57±0.68 | 1.68±0.57 | -0.89±0.58 | <.0001 | 2.32±0.58 | 1.75±0.53 | -0.57±0.52 | <.0001 | 0.0067 |
| **Sebum secretion** | **Total** | 4.92±2.10 | 2.43±1.31 | -2.49±1.52 | <.0001 | 4.57±1.52 | 3.16±1.19 | -1.41±1.02 | <.0001 | <.0001 |
|  | **T-zone** | 5.49±1.94 | 2.63±1.21 | -2.85±1.51 | <.0001 | 4.83±1.58 | 3.33±1.17 | -1.50±1.09 | <.0001 | <.0001 |
|  | **Hairline** | 5.75±1.94 | 2.78±1.41 | -2.97±1.55 | <.0001 | 4.94±1.34 | 3.43±1.03 | -1.51±1.08 | <.0001 | <.0001 |
|  | **Cheek** | 5.33±1.94 | 2.62±1.29 | -2.72±1.51 | <.0001 | 4.63±1.53 | 3.18±1.16 | -1.45±1.01 | <.0001 | <.0001 |
|  | **Jawline** | 4.88±2.17 | 2.41±1.34 | -2.47±1.64 | <.0001 | 4.62±1.65 | 3.11±1.23 | -1.51±1.10 | <.0001 | 0.0023 |
| **Erythema** | **Total** | 2.41±0.68 | 1.68±0.47 | -0.73±0.56 | <.0001 | 2.33±0.60 | 1.71±0.47 | -0.62±0.59 | <.0001 | 0.1058 |
|  | **T-zone** | 2.52±0.64 | 1.78±0.41 | -0.74±0.56 | <.0001 | 2.36±0.60 | 1.74±0.46 | -0.63±0.58 | <.0001 | 0.2746 |
|  | **Hairline** | 2.61±0.67 | 1.81±0.39 | -0.80±0.56 | <.0001 | 2.37±0.59 | 1.84±0.40 | -0.53±0.53 | <.0001 | 0.0224 |
|  | **Cheek** | 2.50±0.65 | 1.76±0.43 | -0.74±0.58 | <.0001 | 2.38±0.58 | 1.75±0.45 | -0.62±0.58 | <.0001 | 0.1869 |
|  | **Jawline** | 2.51±0.65 | 1.68±0.47 | -0.83±0.52 | <.0001 | 2.38±0.62 | 1.71±0.45 | -0.67±0.61 | <.0001 | 0.1336 |
| **Desquamation** | **Total** | 1.64±0.63 | 1.09±0.29 | -0.55±0.55 | <.0001 | 1.55±0.60 | 1.10±0.29 | -0.46±0.55 | <.0001 | 0.1200 |
|  | **T-zone** | 1.68±0.66 | 1.12±0.32 | -0.56±0.57 | <.0001 | 1.53±0.61 | 1.09±0.29 | -0.44±0.58 | <.0001 | 0.2233 |
|  | **Hairline** | 1.75±0.67 | 1.13±0.34 | -0.62±0.57 | <.0001 | 1.61±0.64 | 1.07±0.26 | -0.54±0.61 | <.0001 | 0.4996 |
|  | **Cheek** | 1.71±0.63 | 1.09±0.29 | -0.61±0.55 | <.0001 | 1.57±0.59 | 1.11±0.31 | -0.47±0.53 | <.0001 | 0.0869 |
|  | **Jawline** | 1.67±0.69 | 1.11±0.31 | -0.56±0.58 | <.0001 | 1.57±0.59 | 1.11±0.31 | -0.46±0.55 | <.0001 | 0.3540 |
| **Dryness** | **Total** | 1.69±0.75 | 1.43±0.50 | -0.26±0.72 | <.0001 | 1.62±0.64 | 1.27±0.49 | -0.35±0.62 | <.0001 | 0.2300 |
|  | **T-zone** | 1.51±0.66 | 1.34±0.48 | -0.17±0.69 | 0.0154 | 1.53±0.61 | 1.24±0.50 | -0.29±0.60 | <.0001 | 0.2397 |
|  | **Hairline** | 1.57±0.74 | 1.41±0.49 | -0.16±0.70 | 0.0618 | 1.50±0.61 | 1.21±0.45 | -0.29±0.59 | <.0001 | 0.3084 |
|  | **Cheek** | 1.66±0.75 | 1.43±0.50 | -0.23±0.74 | 0.0005 | 1.65±0.64 | 1.28±0.50 | -0.37±0.62 | <.0001 | 0.1052 |
|  | **Jawline** | 1.86±0.77 | 1.50±0.50 | -0.36±0.75 | <.0001 | 1.80±0.64 | 1.32±0.52 | -0.48±0.61 | <.0001 | 0.3404 |
| **Itching** | **Total** | 1.81±0.64 | 1.07±0.26 | -0.74±0.63 | <.0001 | 1.84±0.59 | 1.11±0.31 | -0.73±0.57 | <.0001 | 0.8740 |
|  | **T-zone** | 1.86±0.61 | 1.07±0.25 | -0.79±0.60 | <.0001 | 1.86±0.61 | 1.10±0.31 | -0.76±0.59 | <.0001 | 0.7213 |
|  | **Hairline** | 1.88±0.61 | 1.09±0.28 | -0.80±0.63 | <.0001 | 1.90±0.59 | 1.10±0.30 | -0.80±0.53 | <.0001 | 0.9793 |
|  | **cheek** | 1.84±0.65 | 1.08±0.27 | -0.76±0.64 | <.0001 | 1.83±0.60 | 1.11±0.32 | -0.71±0.58 | <.0001 | 0.5548 |
|  | **Jawline** | 1.82±0.69 | 1.09±0.28 | -0.73±0.66 | <.0001 | 1.86±0.60 | 1.11±0.31 | -0.75±0.60 | <.0001 | 0.8797 |
| **Tingling sensation** | **Total** | 1.60±0.71 | 1.04±0.19 | -0.56±0.67 | <.0001 | 1.58±0.69 | 1.05±0.23 | -0.52±0.61 | <.0001 | 0.6200 |
|  | **T-zone** | 1.67±0.76 | 1.05±0.22 | -0.62±0.70 | <.0001 | 1.59±0.67 | 1.05±0.22 | -0.54±0.61 | <.0001 | 0.4782 |
|  | **Hairline** | 1.74±0.80 | 1.07±0.26 | -0.67±0.74 | <.0001 | 1.67±0.74 | 1.06±0.23 | -0.61±0.67 | <.0001 | 0.6980 |
|  | **cheek** | 1.67±0.74 | 1.05±0.21 | -0.62±0.69 | <.0001 | 1.60±0.70 | 1.06±0.23 | -0.54±0.62 | <.0001 | 0.3932 |
|  | **Jawline** | 1.67±0.77 | 1.06±0.25 | -0.61±0.71 | <.0001 | 1.61±0.73 | 1.07±0.26 | -0.54±0.63 | <.0001 | 0.5466 |
| **Burning sensation** | **Total** | 1.48±0.67 | 1.03±0.16 | -0.46±0.63 | <.0001 | 1.44±0.69 | 1.03±0.16 | -0.41±0.64 | <.0001 | 0.4871 |
|  | **T-zone** | 1.53±0.70 | 1.03±0.17 | -0.50±0.66 | <.0001 | 1.49±0.70 | 1.01±0.10 | -0.48±0.68 | <.0001 | 0.8361 |
|  | **Hairline** | 1.68±0.76 | 1.06±0.24 | -0.62±0.69 | <.0001 | 1.44±0.67 | 1.01±0.12 | -0.43±0.65 | <.0001 | 0.1143 |
|  | **cheek** | 1.57±0.70 | 1.03±0.18 | -0.54±0.65 | <.0001 | 1.42±0.70 | 1.03±0.18 | -0.39±0.64 | <.0001 | 0.0991 |
|  | **Jawline** | 1.54±0.70 | 1.03±0.18 | -0.51±0.65 | <.0001 | 1.49±0.78 | 1.04±0.19 | -0.45±0.72 | <.0001 | 0.6069 |

Mean±SD(Standard Deviation); Difference= Week 12- Baseline; The type of Investigator-Assessed Sensitivity Scores is Erythema, Desquamation and Desquamation; The type of Patient-Reported Skin Sensitivity Scores is Itching, Tingling and Burning sensation;

*Paired p-value is the wilcoxon signed rank test; **p value of interaction with the retinoid group is result of multiple mixed model adjusted by sex, age, Photo type, Acne type, Prescription Modality

**Supplementary 2.** Change in Quality of Life Scores from Baseline to Week 12, Stratified by Facial Region

| **Quality of Life Scores** | **Age group** | **Total** | | **T-zone** | | **Hairline** | | **cheek** | | **Jawline** | |
| --- | --- | --- | --- | --- | --- | --- | --- | --- | --- | --- | --- |
|  |  | **B (95% CI)** | **p-value** | **B(95% CI)** | **p-value** | **B(95% CI)** | **p-value** | **B(95% CI)** | **p-value** | **B(95% CI)** | **p-value** |
| **Q1** | **Total** | 0.79 (0.71-0.87) | <.0001 | 0.82(0.71-0.93) | <.0001 | 0.93(0.81-1.05) | <.0001 | 0.94(0.86-1.02) | <.0001 | 0.86(0.75-0.97) | <.0001 |
|  | **19 years and under** | 0.77(0.59-0.96) | <.0001 | 0.84(0.64-1.04) | <.0001 | 0.86(0.64-1.09) | <.0001 | 0.86(0.67-1.06) | <.0001 | 0.93(0.60-1.26) | <.0001 |
|  | **20-34 years** | 0.80(0.70-0.91) | <.0001 | 0.83(0.69-0.97) | <.0001 | 1.00(0.85-1.15) | <.0001 | 0.94(0.83-1.05) | <.0001 | 0.87(0.72-1.02) | <.0001 |
|  | **Over 35 years** | 0.76(0.57-0.94) | <.0001 | 0.69(0.25-1.13) | 0.0045 | 0.50(-0.15-1.15) | 0.1106 | 1.03(0.80-1.27) | <.0001 | 0.79(0.58-0.99) | <.0001 |
| **Q2** | **Total** | 1.00(0.92-1.09) | <.0001 | 1.08(0.96-1.19) | <.0001 | 1.12(0.99-1.25) | <.0001 | 1.11(1.01-1.20) | <.0001 | 1.10(0.99-1.22) | <.0001 |
|  | **19 years and under** | 1.05(0.84-1.25) | <.0001 | 1.20(0.98-1.41) | <.0001 | 1.11(0.86-1.37) | <.0001 | 1.10(0.89-1.32) | <.0001 | 1.25(0.94-1.56) | <.0001 |
|  | **20-34 years** | 0.98(0.87-1.09) | <.0001 | 1.06(0.92-1.19) | <.0001 | 1.13(0.97-1.28) | <.0001 | 1.08(0.97-1.20) | <.0001 | 1.06(0.91-1.22) | <.0001 |
|  | **Over 35 years** | 1.04(0.85-1.23) | <.0001 | 0.81(0.36-1.26) | 0.0016 | 1.00(0.20-1.80) | 0.0224 | 1.26(1.02-1.50) | <.0001 | 1.10(0.89-1.30) | <.0001 |
| **Q3** | **Total** | 0.08(0.05-0.12) | <.0001 | 0.11(0.05-0.17) | 0.0007 | 0.08(0.01-0.15) | 0.0354 | 0.10(0.04-0.15) | 0.0003 | 0.07(0.01-0.13) | 0.0195 |
|  | **19 years and under** | 0.14(0.03-0.25) | 0.0156 | 0.13(0.01-0.24) | 0.0287 | 0.11(-0.02-0.25) | 0.0938 | 0.15(0.03-0.27) | 0.0152 | 0.07(-0.07-0.21) | 0.3001 |
|  | **20-34 years** | 0.07(0.01-0.13) | 0.0200 | 0.10(0.01-0.18) | 0.0225 | 0.05(-0.04-0.13) | 0.3040 | 0.08(0.01-0.14) | 0.0185 | 0.07(-0.01-0.16) | 0.0866 |
|  | **Over 35 years** | 0.06(-0.03-0.15) | 0.1793 | 0.19(-0.11-0.48) | 0.1947 | 0.25(-0.36-0.86) | 0.3559 | 0.10(-0.05-0.24) | 0.1789 | 0.07(-0.03-0.18) | 0.1791 |
| **Q4** | **Total** | 0.73(0.65-0.81) | <.0001 | 0.69(0.58-0.79) | <.0001 | 0.71(0.61-0.81) | <.0001 | 0.94(0.87-1.01) | <.0001 | 0.79(0.69-0.89) | <.0001 |
|  | **19 years and under** | 0.70(0.53-0.87) | <.0001 | 0.71(0.53-0.90) | <.0001 | 0.68(0.50-0.86) | <.0001 | 0.83(0.69-0.97) | <.0001 | 0.82(0.63-1.01) | <.0001 |
|  | **20-34 years** | 0.76(0.65-0.86) | <.0001 | 0.71(0.58-0.84) | <.0001 | 0.77(0.65-0.89) | <.0001 | 0.96(0.87-1.04) | <.0001 | 0.81(0.67-0.94) | <.0001 |
|  | **Over 35 years** | 0.69(0.47-0.92) | <.0001 | 0.44(0.07-0.80) | 0.0227 | 0.25(-0.15-0.65) | 0.1775 | 1.10(0.87-1.33) | <.0001 | 0.71(0.48-0.95) | <.0001 |
| **Q5** | **Total** | 1.02(0.94-1.11) | <.0001 | 0.99(0.88-1.10) | <.0001 | 0.94(0.82-1.07) | <.0001 | 1.16(1.06-1.25) | <.0001 | 1.08(0.97-1.20) | <.0001 |
|  | **19 years and under** | 0.95(0.76-1.15) | <.0001 | 1.07(0.87-1.27) | <.0001 | 0.93(0.70-1.16) | <.0001 | 1.00(0.80-1.20) | <.0001 | 0.89(0.57-1.22) | <.0001 |
|  | **20-34 years** | 1.06(0.95-1.18) | <.0001 | 1.01(0.87-1.15) | <.0001 | 0.99(0.84-1.14) | <.0001 | 1.18(1.06-1.30) | <.0001 | 1.16(1.01-1.31) | <.0001 |
|  | **Over 35 years** | 0.96(0.73-1.18) | <.0001 | 0.56(0.18-0.95) | 0.0073 | 0.50(-0.15-1.15) | 0.1106 | 1.32(1.06-1.59) | <.0001 | 1.02(0.78-1.27) | <.0001 |
| **SUM** | **Total** | 3.63(3.38-3.88) | <.0001 | 3.69(3.33-4.04) | <.0001 | 3.78(3.41-4.15) | <.0001 | 4.24(3.99-4.49) | <.0001 | 3.90(3.56-4.25) | <.0001 |
|  | **19 years and under** | 3.61(3.00-4.21) | <.0001 | 3.95(3.31-4.58) | <.0001 | 3.70(2.99-4.42) | <.0001 | 3.95(3.39-4.51) | <.0001 | 3.96(3.10-4.83) | <.0001 |
|  | **20-34 years** | 3.67(3.33-4.02) | <.0001 | 3.70(3.26-4.14) | <.0001 | 3.93(3.50-4.36) | <.0001 | 4.24(3.93-4.55) | <.0001 | 3.97(3.51-4.44) | <.0001 |
|  | **Over 35 years** | 3.51(2.86-4.16) | <.0001 | 2.69(1.18-4.19) | 0.0017 | 2.50(-0.24-5.24) | 0.0667 | 4.81(4.09-5.53) | <.0001 | 3.69(2.99-4.39) | <.0001 |

Type of Quality of Life Scores is Q1-Q5; Q1. As a result of having acne, during the last month have you been aggressive, frustrated or embarrassed?; Q2. Do you think that having acne during the last month interfered with your daily social life, social events or relationships with members of the opposite sex?; Q3. During the last month have you avoided public changing facilities or wearing swimming costumes because of your acne?; Q4. How would you describe your feelings about the appearance of your skin over the last month?; Q5. Please indicate how bad you think your acne is now; SUM. Sum of Quality of Life Scores(Q1+Q2+Q3+Q4+Q5)

*p value of interaction between Baseline and Week 12 is result of multiple mixed model adjusted by sex, age, Photo type, Acne type, Prescription Modality;

**Supplementary 3.** Change in Quality of Life Scores from Baseline to Week 12

| **Q1** | **Q2** | **Q3** |
| --- | --- | --- |
| 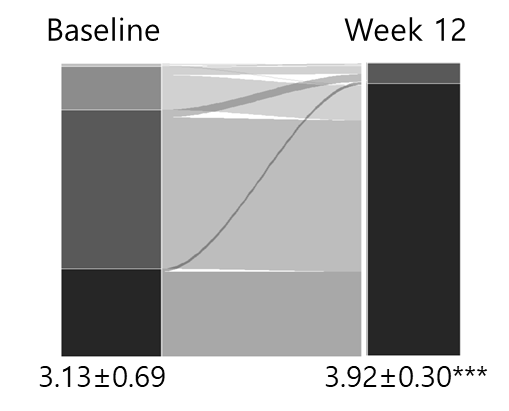 | 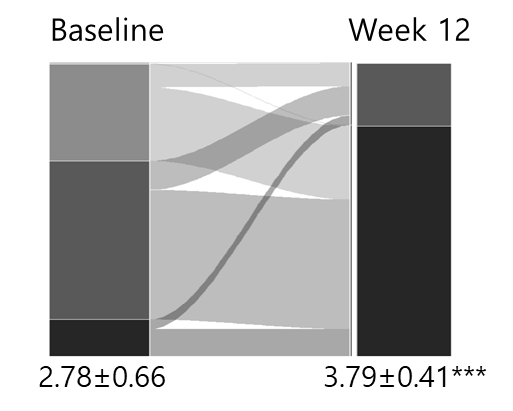 | 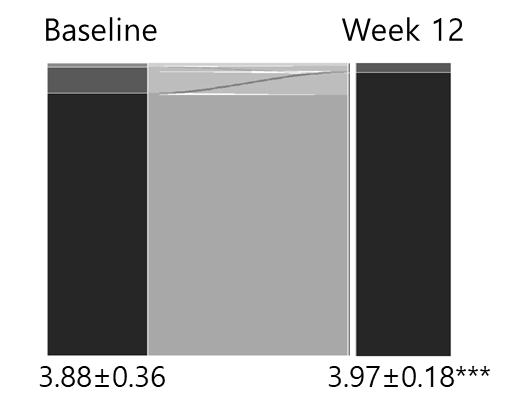 |
| **Q4** | **Q5** |  |
| 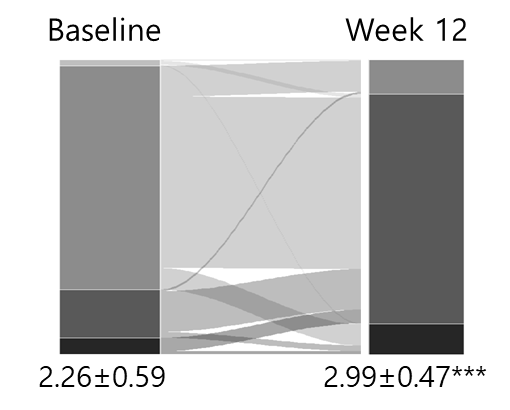 | 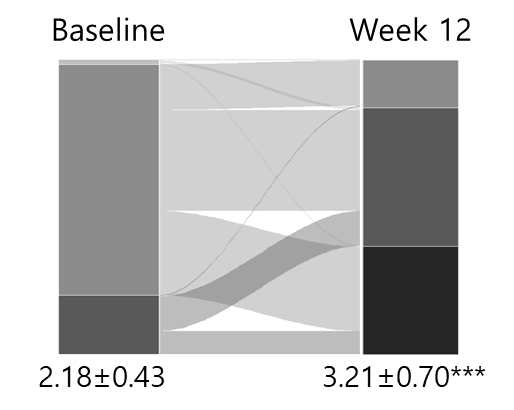 | 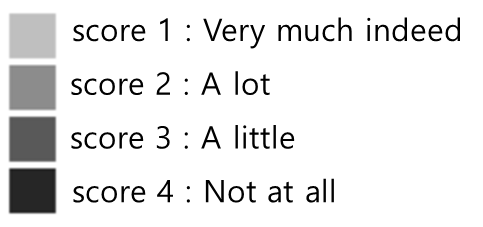 |

Alluvial plot; Mean±SD(Standard Deviation) of score; Type of Quality of Life Scores is Q1-Q5; Q1. As a result of having acne, during the last month have you been aggressive, frustrated or embarrassed?; Q2. Do you think that having acne during the last month interfered with your daily social life, social events or relationships with members of the opposite sex?; Q3. During the last month have you avoided public changing facilities or wearing swimming costumes because of your acne?; Q4. How would you describe your feelings about the appearance of your skin over the last month?; Q5. Please indicate how bad you think your acne is now;

*** p value of interaction between Baseline and Week 12 is result of multiple mixed model adjusted by sex, age, Photo type, Acne type, Prescription Modality; p value is <0.0001
